# Response to: Flavia Magazoni Gonçalves, Marcello Rizzatti Luizon, Jose G. Speciali: “Haplotypes in candidate genes related to nitric oxide pathways and vascular permeability associated with migraine with aura”

**DOI:** 10.1007/s10194-012-0446-5

**Published:** 2012-04-12

**Authors:** Markus Schürks

**Affiliations:** Department of Neurology, University Hospital Essen, Hufelandstrasse 55, 45122 Essen, Germany

Migraine is regarded as a “neurovascular disorder” presenting with dysfunctions of both the brain and the vascular system. However, the initial spark causing migraine appears to be generated within the brain, which is supported by results from recent genome-wide association studies (GWAS) [1, 2], while vascular phenomena are likely epiphenomena [3]. In addition, migraine with aura has been associated with an increased risk for ischemic stroke, while such an association is less clear for other vascular disorders [4]. In contrast, migraine without aura, which is far more common than migraine with aura, does not seem to carry an increased risk for vascular disease.

So, how are the brain and the vasculature linked in migraine? The short answer is: We do not really know. A longer answer would first have to state that the link is likely complex and may include shared genetics. One approach to decipher this link is to investigate variants in genes involved in vascular tone control, including genes coding for the nitric oxide synthases (NOS), a family of enzymes found in the nervous system, cardiovascular system, and endothelium [5]. Results at the single variant level from studies investigating *NOS2* and *NOS3* genes [6–11] as well as the vascular endothelial growth factor (*VEGF*) gene [12] in migraine did not give a clear answer. Reasons may include the wide phenotypic spectrum of migraine, differences in study design, genetically heterogeneous populations, and limited sample sizes. Analyses utilizing the integrated information from multiple gene variants such as haplotype analyses may be beneficial as suggested by recent studies [10–12]. However, it also has to be taken into consideration that studies using even more comprehensive and more efficient approaches like GWAS failed to identify any vascular-related genes among migraineurs [1, 2, 13].

What we have learned from studying migraine genetics so far? First, migraine is clinically and genetically heterogeneous making it notoriously difficult to identify susceptibility genes. Any susceptibility gene variant will modify the risk for migraine to only a small to moderate degree; hence, large studies are needed to detect them. Second, any association found—whatever study design used—must be replicated in independent samples/cohorts to make us confident that the associations seen are true and not spurious. Third, novel ways of analyses, for example, investigating the interaction of gene variants may help to understand why and how certain variants influence migraine pathophysiology. However, in order to avoid spurious results interaction analyses should only be performed among gene variants for which there is strong evidence of an association with migraine and if there is enough power to really detect potential interactions.
